# Integrity of the Anterior Visual Pathway and Its Association with Ambulatory Performance in Multiple Sclerosis

**DOI:** 10.1155/2013/481035

**Published:** 2013-06-24

**Authors:** Swathi Balantrapu, Brian M. Sandroff, John H. Pula, Robert W. Motl

**Affiliations:** ^1^Department of Kinesiology and Community Health, University of Illinois at Urbana-Champaign, 233 Freer Hall, Urbana, IL 61801, USA; ^2^College of Medicine, University of Illinois at Peoria, Peoria, IL 61656, USA

## Abstract

*Background*. Retinal nerve fiber layer thickness (RNFLT) and total macular volume (TMV) represent markers of neuroaxonal degeneration within the anterior visual pathway that might correlate with ambulation in persons with multiple sclerosis (MS). *Objective*. This study examined the associations between RNFLT and TMV with ambulatory parameters in MS. *Methods*. Fifty-eight MS patients underwent a neurological examination for generation of an expanded disability status scale (EDSS) score and measurement of RNFLT and TMV using optical coherence tomography (OCT). Participants completed the 6-minute walk (6MW) and the timed 25-foot walk (T25FW). The associations were examined using generalized estimating equation models that accounted for within-patient, inter-eye correlations, and controlled for disease duration, EDSS score, and age. *Results*. RNFLT was not significantly associated with 6MW (*P* = 0.99) or T25FW (*P* = 0.57). TMV was significantly associated with 6MW (*P* = 0.023) and T25FW (*P* = 0.005). The coefficients indicated that unit differences in 6MW (100 feet) and T25FW (1 second) were associated with 0.040 and −0.048 unit differences in TMV (mm^3^), respectively. *Conclusion*. Integrity of the anterior visual pathway, particularly TMV, might represent a noninvasive measure of neuroaxonal degeneration that is correlated with ambulatory function in MS.

## 1. Introduction

The anterior visual pathway (i.e., retina, optic nerves, chiasm, and optic tracts) represents a valuable model for understanding axonal and neuronal loss and clinical and functional correlates in persons with multiple sclerosis (MS) [1]. This pathway, particularly the optic nerve, is often affected by the MS pathological disease processes given its proximity with the vasculature around the ventricles of the brain [2]. The integrity of the anterior visual pathway can be noninvasively imaged using optical coherence tomography (OCT) of the retina. OCT provides metrics of retinal nerve fiber layer thickness (RNFLT) and total macular volume (TMV). RNFLT reflects the integrity of nonmyelinated axonal tissue, whereas TMV reflects the integrity of nonmyelinated axonal tissue as well as all retinal layers including cellular segments [3]. We further note that RNFLT and TMV have been associated with lesion volume and brain atrophy metrics from magnetic resonance imaging in persons with MS [4–6]. This is important as imaging the anterior visual pathway may provide a correlate of the clinical and functional consequences of axonal and neuronal loss in MS.

The integrity of the anterior visual pathway might be associated with walking function in persons with MS. Visual and ambulatory functions are integrally related in all persons, including those with MS [7], and represent two of the most valued functions that are compromised in the MS population [7–9]. Importantly, OCT metrics have been associated with the expanded disability status scale (EDSS) scores in persons with MS (range: *r* = −0.30 through −0.70) [10], and the EDSS is heavily weighted by ambulatory function in the middle range of scores based on 500-meter walk performance [11]. OCT metrics have further been correlated with other measures of disability such as the multiple sclerosis functional composite (MSFC) (*r* = 0.227) [12], and the MSFC has an ambulatory component based on the timed 25-foot walk (T25FW). Lastly, RNFLT and TMV metrics from OCT might reflect neurodegeneration on a more global level [13], thus supporting a possible association with walking impairment—another global manifestation of MS [14].

The motivation for considering a possible association between integrity of the anterior visual pathway and walking function is further rooted in the idea that OCT metrics can serve as structural outcomes for trials examining neuroprotective and neuroreparative outcomes in MS [15]. To that end, many rehabilitation trials, for example, clinical trials of exercise training, are targeted towards improving ambulatory function in MS. Such a beneficial effect might have a basis in structural adaptations involving axons and neurons [16, 17]. If true, then OCT and its metrics might be considered for inclusion in clinical trials of rehabilitation for capturing the possible influence on central nervous system (CNS) integrity in MS.

This study examined the association between integrity of the anterior visual pathway and walking functions in persons with MS. We hypothesized that the metrics of RNFLT and TMV would be associated with scores on well-characterized walking outcomes, namely, the six-minute walk (6MW) and the T25FW [14, 18], even after controlling for possible covariates (e.g., disease duration, EDSS, and age).

## 2. Methods

### 2.1. Participants

We contacted and screened 113 possible participants residing in Central Illinois between the middle of September, 2010, through the last week of December, 2010. The possible participants were not consecutive patients, but were referrals from the practices of three locally residing neurologists as well as our laboratory database. The final sample consisted of 58 patients with a definite diagnosis of MS who satisfied our inclusion criteria. The criteria for inclusion were the following: (a) capacity for independent ambulation or ambulation with an assistive device (e.g., cane, crutch, walking frame, or rollator walker); (b) relapse free during the previous 30-day period before testing; and (c) willingness to undergo testing. We excluded persons when conducting OCT who had (a) ocular disease (prior history of optic neuritis, glaucoma, macular degeneration, etc.) or (b) high myopia (minus 7.5 or higher) of both eyes based on a clinical examination from a neuro-ophthalmologist (John H. Pula).

### 2.2. Primary Measures

#### 2.2.1. OCT

Participants underwent time-domain OCT scans (Zeiss Stratus OCT 3, Carl Zeiss Meditec, Dublin, CA, USA) performed by a neuro-ophthalmologist (John H. Pula). When necessary, 1% phenylephrine intraocular mydriatic drops were instilled prior to scanning. Throughout scanning, the patient was fixated on an internal target provided by the equipment. Each subject eye underwent sequential FAST RNFLT circle scan and FAST macular scan protocols. If a scan had poor image quality, defined as improper disc centering, poor reference image acquisition, or a signal strength score of less than 7, it was repeated until obtaining an adequate scan. Average RNFLT was determined as the distance between the first reflection from the vitreoretinal interface and the anterior boundary of the second reflective layer, corresponding to Bruch's Membrane, and average TMV was recorded as an overall macular volume which did not contain the optic nerve head.

#### 2.2.2. 6MW

The 6MW was performed in a rectangular corridor with hallways that exceed 50 m in length and that were clear of obstructions and foot traffic. We provided standardized instructions and emphasized walking as far and as fast as possible for 6 minutes [19]. One researcher followed alongside of the participant for safety, while another researcher followed 3–5 feet behind the participant and recorded the distance travelled in feet using a measuring wheel (Stanley MW50, New Britain, CT, USA) [20].

#### 2.2.3. T25FW

The T25FW was performed along a clearly marked 25-foot long path on a carpeted corridor that was clear of obstructions and foot traffic. We provided standardized instructions and emphasized walking as fast and as safely as possible [21]. One researcher followed alongside the participant for safety, and another recorded the time in seconds by using a stopwatch. The T25FW was performed twice, and the average of the two trials was included for the analysis.

#### 2.2.4. EDSS

Participants underwent a neurological exam for generation of an EDSS score. EDSS scoring includes tests of 8 different functional systems (FS), including visual, brainstem, pyramidal, cerebellar, sensory, bowel/bladder, cerebral, and ambulatory function. Each of these separate functional system scores received a “step” score, and, at the end, all “step” scores were combined with gait function (500-meter walk) into an overall EDSS score. EDSS scores range from 0 (no disability) to 10 (death from MS) [22].

### 2.3. Procedure

The procedure was approved by a university institutional review board (i.e., the United States equivalent of an ethics review committee), and all participants provided written informed consent. The measures were administered in a single session by the trained and experienced staff of an MS research center. The participants provided demographic information and underwent OCT scanning and a neurological examination for generating the EDSS score. The participants further performed the 6MW and the T25FW. All participants received $15 remuneration for travel expenses.

### 2.4. Data Analysis

The data were analyzed using SPSS version 18 (IBM, Chicago, IL, USA). We provided descriptive statistics (e.g., mean, median, standard deviation (SD), and range of scores) for the demographic, clinical, OCT, and ambulatory outcomes. We examined the associations between OCT metrics (RNFLT and TMV) with ambulatory outcomes (6MW and T25FW performance) using generalized estimating equation (GEE) models. The GEE models were adjusted for within-patient, inter-eye correlations, and controlled for age, disease duration, and EDSS scores when examining the linear associations between OCT metrics and ambulatory outcomes. The alpha for statistical significance was set at 0.05.

## 3. Results

### 3.1. Sample Characteristics

Participants were ambulatory women (*n* = 46) and men (*n* = 12) with a definite diagnosis of MS. The mean (SD) age was 52 (11) years, and the sample primarily had a relapsing-remitting clinical course (*n* = 44; 76%). The mean (SD) disease duration was 11 (10) years, and the sample had a median EDSS score of 4.5 (interquartile range = 2.0).

### 3.2. Descriptive Statistics

The data for RNFLT, TMV, T25FW, and 6MW are provided in Table 1. The mean (SD) values for RNFLT and TMV of persons (without optic neuritis or any other ocular diseases) in the present study were similar to those in a previous research [23]. The mean (SD) values for T25FW and 6MW were consistent with those in previous researches [19, 24].

### 3.3. Associations between OCT Metrics and Ambulatory Outcomes

**Figure 1:**
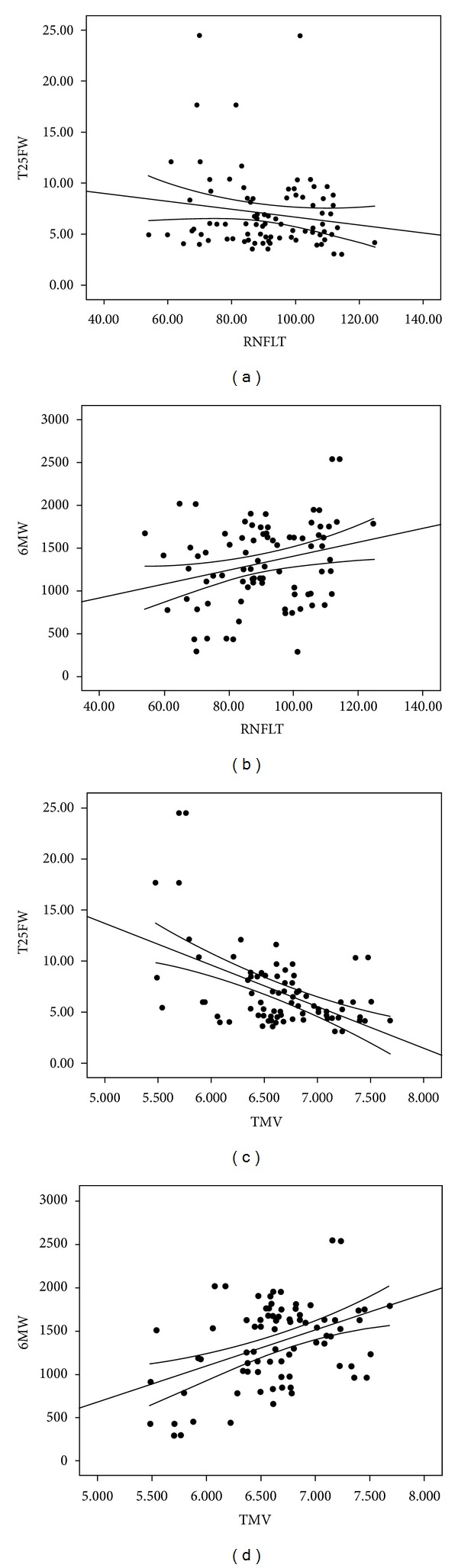
Scatter plots with linear trend lines and 95% confidence intervals for associations between retinal nerve fiber layer thickness (RNFLT) and total macular volume (TMV) with timed 25-foot walk (T25FW) and six-minute walk (6MW) performance.

The GEE models accounting for inter-eye correlations and age, disease duration, and EDSS scores indicated that RNFLT was not significantly associated with 6MW (*P* = 0.99) or T25FW (*P* = 0.57). By comparison, the GEE models accounting for inter-eye correlations and age, disease duration, and EDSS scores indicated that TMV was significantly associated with 6MW (*P* = 0.023) and T25FW (*P* = 0.005). The coefficients indicated that unit differences in 6MW (100 feet) and T25FW (1 second) were associated with 0.040 and −0.048 unit differences in TMV (mm^3^), respectively. Scatter plots of associations between RNFLT and TMV with T25FW and 6MW are provided in Figure 1. These scatter plots confirm the presence of weak correlations between RNFLT with T25FW and 6MW, but stronger correlations between TMV and the ambulatory outcomes.

## 4. Discussion

The present study demonstrated that integrity of anterior visual pathways based on OCT metrics was associated with ambulatory outcomes in persons with MS. Those who had reduced TMV, in particular, walked a shorter distance during the 6MW and slower during the T25FW, independent of disease duration, EDSS, and age in the GEE models. Collectively, this research indicates that OCT metrics of anterior visual pathway integrity, particularly TMV, are associated with functional consequences, namely, reduced ambulatory performance, in MS. This might position OCT and its metrics as an important outcome for inclusion within rehabilitation research of walking function; such metrics might provide indications of possible neuroprotective and neuroreparative consequences of rehabilitation based on integrity of the anterior visual pathway.

Researchers have previously reported associations between OCT metrics and EDSS as well as MSFC scores in persons with MS [19, 25]. This is important as both the EDSS and MSFC include ambulatory measures of the 500-meter walk and T25FW, respectively, when generating an overall score. Such observations, in part, motivated our interest in examining the associations between RNFLT and TMV with ambulatory outcomes, and controlling for covariates, particularly EDSS scores. To that end, our results indicate an association between TMV and both 6MW and T25FW performance, independent of EDSS as well as age and MS duration. Accordingly, this is the first study, to our knowledge, that has reported an independent association between OCT metrics of anterior visual pathway integrity and ambulatory performance in persons with MS.

The mean values of RNFLT, TMV, T25FW, and 6MW of persons with MS (without optic neuritis or any other ocular diseases) in the present study were consistent with those in previous researches [19, 23, 24]. For example, the mean (SD) RNFLT in the present study was 90.9 (14.9) *μ*m, and this was consistent with the value of 95.6 (14.5) *μ*m from a previous research [23]. The same consistency in mean values is seen with TMV [23]. Regarding ambulatory function, the T25FW and 6MW values of 6.9 (3.5) seconds and 1,336 (441) feet, respectively, are consistent with the values of 6.4 (2.7) and 1,277 (255) in previous researches [19, 24]. Such observations are important for contextualizing our sample and results within the broader research on OCT metrics and ambulatory function involving persons with MS.

The integrity of the anterior visual pathway might be associated with ambulatory performance as vision is coupled with walking. Indeed, vision and gait are two of the most important bodily functions that are compromised, yet highly valued, across the early and late phases of MS [7]. Visual dysfunction has further been identified as one of several possible impairments (e.g., weakness, sensory loss, and ataxia) that might influence walking in neurological diseases including MS [9]. Overall, we are not surprised that integrity of the anterior visual pathway is a correlate of walking—another global indicator of MS progression—and this study provides the first data supporting the presumed association between visual and walking functions in MS [9].

We believe that one possible implication of our study is that OCT metrics might be included in rehabilitation trials for understanding adaptations within the CNS that correspond with improvements in ambulatory function. Indeed, rehabilitation interventions that improve gait in MS might do so, in part, through neuroreparative and neuroprotective processes. If correct, then OCT metrics might be considered for inclusion in clinical trials of rehabilitation approaches for improving ambulation in people with MS. Such an approach provides simple, affordable, and noninvasive metrics for documenting possible neuroreparative and neuroprotective outcomes.

There are many strengths of the current study including the first examination of anterior visual pathway metrics and walking function controlling for EDSS and other covariates in MS. There are important limitations of the current study. The most important limitation is that the study included time-domain OCT, and this has poor resolution and reduced accuracy of outcomes. This would seemingly bias any associations with other outcomes towards the null and could be overcome in future research using spectral domain OCT and better characterizing associations with ambulatory outcomes. We do not have data from spectral domain OCT for addressing this limitation in the current study. The sample size might be considered small, but it is sufficient for a preliminary examination of OCT metrics and ambulation. The sample mostly had relapsing-remitting MS, and this limits the generalizability of our data and results amongst those with progressive MS. The study included a cross-sectional design and did not provide data on changes in OCT metrics and ambulatory outcomes over time. Lastly, the T25FW was performed on a carpeted surface, and this differs from the typical administration on a noncarpeted walkway [21]. This might have reduced walking speed on the T25FW. Despite those limitations, we are excited by the novel data indicating that OCT metrics of anterior visual pathway integrity are associated with walking outcomes in MS.

## Figures and Tables

**Table 1:** Mean, standard deviation, and range of values for optical coherence tomography metrics and ambulatory outcomes.

Variable	Mean	Standard deviation	Range
TMV (mm^3^)	6.63	0.49	5.49–7.68
RNFLT (*μ*m)	90.9	14.9	5.41–124.8
6MW (feet)	1,336	441	294–2,538
T25FW (seconds)	6.9	3.5	3.1–24.5

Note. TMV: total macular volume. RNFLT: retinal nerve fiber layer thickness. 6MW: six-minute walk. T25FW: timed 25-foot walk.

## References

[1] Galetta KM, Calabresi PA, Frohman EM, Balcer LJ (2011). Optical coherence tomography (OCT): imaging the visual pathway as a model for neurodegeneration. *Neurotherapeutics*.

[2] Schmitt C, Strazielle N, Ghersi-Egea JF (2012). Brain leukocyte infiltration initiated by peripheral inflammation or experimental autoimmune encephalomyelitis occurs through pathways connected to the CSF-filled compartments of the forebrain and midbrain. *Journal of Neuroinflammation*.

[3] Oberwahrenbrock T, Schippling S, Ringelstein M (2012). Retinal damage in multiple sclerosis disease subtypes measured by high-resolution optical coherence tomography. *Multiple Sclerosis International*.

[4] Dörr J, Wernecke KD, Bock M (2011). Association of retinal and macular damage with brain atrophy in multiple sclerosis. *PLoS One*.

[5] Frohman EM, Dwyer MG, Frohman T (2009). Relationship of optic nerve and brain conventional and non-conventional MRI measures and retinal nerve fiber layer thickness, as assessed by OCT and GDx: a pilot study. *Journal of the Neurological Sciences*.

[6] Siger M, Dziȩgielewski K, Jasek L (2008). Optical coherence tomography in multiple sclerosis: thickness of the retinal nerve fiber layer as a potential measure of axonal loss and brain atrophy. *Journal of Neurology*.

[7] Heesen C, Böhm J, Reich C, Kasper J, Goebel M, Gold SM (2008). Patient perception of bodily functions in multiple sclerosis: gait and visual function are the most valuable. *Multiple Sclerosis*.

[8] Compston A, Coles A (2008). Multiple sclerosis. *The Lancet*.

[9] Pearson OR, Busse ME, van Deursen RWM, Wiles CM (2004). Quantification of walking mobility in neurological disorders. *QJM*.

[10] Petzold A, de Boer JF, Schippling S (2010). Optical coherence tomography in multiple sclerosis: a systematic review and meta-analysis. *The Lancet Neurology*.

[11] Goldman MD, Motl RW, Rudick RA (2010). Possible clinical outcome measures for clinical trials in patients with multiple sclerosis. *Therapeutic Advances in Neurological Disorders*.

[12] Toledo J, Sepulcre J, Salinas-Alaman A (2008). Retinal nerve fiber layer atrophy is associated with physical and cognitive disability in multiple sclerosis. *Multiple Sclerosis*.

[13] Gordon-Lipkin E, Chodkowski B, Reich DS (2007). Retinal nerve fiber layer is associated with brain atrophy in multiple sclerosis. *Neurology*.

[14] Motl RW (2013). Ambulation and multiple sclerosis. *Physical Medicine and Rehabilitation Clinics of North America*.

[15] Barkhof F, Calabresi PA, Miller DH, Reingold SC (2009). Imaging outcomes for neuroprotection and repair in multiple sclerosis trials. *Nature Reviews Neurology*.

[16] Rossi S, Furlan R, de Chiara V (2009). Exercise attenuates the clinical, synaptic and dendritic abnormalities of experimental autoimmune encephalomyelitis. *Neurobiology of Disease*.

[17] Hessen C, Romberg A, Gold S, Schulz K-H (2006). Physical exercise in multiple sclerosis: supportive care or a putative disease-modifying treatment. *Expert Review of Neurotherapeutics*.

[18] Kieseier BC, Pozzilli C (2012). Assessing walking disability in multiple sclerosis. *Multiple Sclerosis*.

[19] Goldman MD, Marrie RA, Cohen JA (2008). Evaluation of the six-minute walk in multiple sclerosis subjects and healthy controls. *Multiple Sclerosis*.

[20] Motl RW, Dlugonski D, Suh Y, Weikert M, Fernhall B, Goldman M (2010). Accelerometry and its association with objective markers of walking limitations in ambulatory adults with multiple sclerosis. *Archives of Physical Medicine and Rehabilitation*.

[21] Fischer JS, Rudick RA, Cutter GR, Reingold SC (1999). The Multiple Sclerosis Functional Composite Measure (MSFC): an integrated approach to MS clinical outcome assessment. National MS Society Clinical Outcomes Assessment Task Force. *Multiple Sclerosis*.

[22] Kurtzke JF (1983). Rating neurologic impairment in multiple sclerosis: an expanded disability status scale (EDSS). *Neurology*.

[23] Burkholder BM, Osborne B, Loguidice MJ (2009). Macular volume determined by optical coherence tomography as a measure of neuronal loss in multiple sclerosis. *Archives of Neurology*.

[24] Phan-Ba R, Calay P, Grodent P (2012). Motor fatigue measurement by distance-induced slow down of walking speed in multiple sclerosis. *PLoS One*.

[25] Albrecht P, Fröhlich R, Hartung H-P, Kieseier BC, Methner A (2007). Optical coherence tomography measures axonal loss in multiple sclerosis independently of optic neuritis. *Journal of Neurology*.

